# A Lecture on Hæmoptysis

**Published:** 1839-02-16

**Authors:** N. Chapman

**Affiliations:** Professor of the Theory and Practice of Physic, in the University of Pennsylvania


### MEDICAL EXAMINER.

DEVOTED TO MEDICINE, SURGERY, AND THE COLLATERAL SCIENCES.

No. 7.] PHILADELPHIA, SATURDAY, FEBRUARY 16, 1839. [Vol. II.

### A LECTURE ON HÆMOPTYSIS.

*By* N. Chapman, M. D., *Professor of the Theory and Practice of Physic, in the University of Pennsylvania.*

(Concluded from page 88.)

In place, however, of the active hæmorrhage, of which I have now disposed, cases are, perhaps, as frequently to be encountered in a very opposite condition of system. Commonly they are found among the valetudinary, and especially those of scrofulous or tubercular tendencies. Manifestations of incipient or more advanced consumption exist in many instances. Together with a dry, diminutive cough, hurried respiration, and more or less pain or uneasiness of chest, we have a quick, irritated, or very feeble pulse, occasional hectic flushes, much prostration of strength, and a pallid or sallow skin, with softness, flaccidity, and bloatedness. The discharge of blood may be small, in sufficient quantity merely to streak the sputa, or, perhaps, a mouthful or two of it, and then ceasing for a time. Extreme laxity of the exhalents existing, or proceeding from congestion of the lungs, it is copious, pouring in a stream, so that a pint or more escapes.

Concerning the cure, there are mainly the same objects to be attained, as in the preceding form of the disease. The first is to check the bleeding when profuse, and to which end, the means before enumerated, except the evacuant, or otherwise depressing, may be employed. Even these, however, are not totally to be excluded under certain circumstances. It may, indeed, become indispensably necessary, where there is very heavy oppression, to take away a small portion of blood, generally or topically.

As an additional remedy, the spirit of turpentine should be mentioned, given in the dose of ten, fifteen, or twenty drops, very frequently repeated. The powdered capsicum, in four or five grains, repeated in the same way, has also been recommended, though its propriety seems to me very doubtful. From a scruple to half a drachm of the nitrate of potash, in an ounce of brandy, is very effectual, according to some recent reports. Neither of these two last remedies have I tried. Cullen praises alum, which I have not found of service,—and the same remark applies to certain vegetable astringents, as kino, catechu, &c. Greater advantage may be derived from the elixir vitriol, ten or fifteen drops at a time, adequately diluted in sweetened water, and much has recently been said of the creosote.

The most decisive of all measures, however, is an emetic, the *modus operandi* of which is not obscure, and the practice may be vindicated *a priori*, independently of any evidence of facts in support of the deduction of reasoning.

In hæmorrhage, there is a want of equilibrium in the circulation, occasioning irregular determinations of blood, some one organ being surcharged at the expense of other portions of the system. The impression of the emetic, in conformity with an old aphorism, "ubi irritatio, ibi affluxus," probably invites, primarily, a current to the stomach as a centre of fluxion, and thereby immediately tends to exonerate the previously affected organ from its oppressive congestion,—and, secondarily, by filling the cutaneous vessels especially, re-distributes the blood, and hence restores that just balance, which had been subverted. Effects like these from puking, are very observable in the congestive forms of fever, and other acute diseases. In our late typhoid epidemics, both of the winter and summer, how effectual this process proved in relieving engorgements of the great viscera, is sufficiently known. Numerous were the instances which I saw myself of its extraordinary success where the liver, or the spleen, or the lungs, or even the brain, was unduly loaded.

But more than I have indicated, is to be ascribed to emetics in restraining hæmorrhage. Nausea itself represses the force of the circulation, and in some cases must be useful,—though it is to the controlling influence over the whole of the capillaries, changing that condition which admits of sanguineous exhalation, that their efficacy is mainly owing. As colliquative perspiration, watery diarrhœa, and hydropic effusion, are sometimes arrested by vomiting, so does it operate in hæmorrhage. The exhalents in all these cases, under certain circumstances, become morbidly changed,—and a sanguineous or serous discharge ensues, according to the peculiar modification of condition which may exist at the time.

It remains only to detail some of the results of my experience, in confirmation of the efficacy of the practice which has been suggested, to be illustrated by a few examples.

In 1807, I was called to a young man of consumptive tendencies, who, for several months, had suffered occasionally from hæmoptysis, and was treated by another physician, in part, by digitalis. Being suddenly attacked with a copious effusion of blood, he took before my visit an exorbitant dose of the medicine, which excited vomiting,—the hæmorrhage ceased, he became convalescent, and ultimately recovered. Effects so decided, I did not then impute altogether to the act of puking. As often happens from digitalis, an extremely distressing nausea continued for several days, to which I thought it probable the permanent benefit was, in a considerable degree, owing.

Encouraged, however, by the cure, and influenced, perhaps, still more by theoretical notions of the nature of hæmorrhage, and of the applicability of the remedy to it, I resolved to subject the practice to a further and fairer trial.

It was not long before I had an opportunity of doing so, in the instance of a young man from the country, whom I had been attending for several weeks, for pulmonary consumption. Three years previously to my seeing him, he was compelled to abandon the study of the law on account of the frequent recurrence of spitting of blood—and when he came under my care, was far advanced in phthisis. One night, he was aroused from sleep by a repetition of the hæmorrhage, and on my arrival had lost more than a pint of blood without any diminution of the flow. Common salt, sugar of lead, and such like articles, were used in vain,—and bleeding, generally or locally, seemed inadmissible, from the debilitated state of the system, and of the pulse especially. Excepting an emetic, I was nearly destitute of resource in this emergency,—and, accordingly, twenty grains of ipecacuanha were administered, which soon bringing on vomiting, the effusion was suppressed. On several subsequent occasions, the same means proved equally effectual, though, ultimately, he died of the main disease.

I had, within a month, a third case, which afforded me a further opportunity of pursuing the practice, and of confirming my confidence in its efficacy. It was that of a young woman, who, a considerable time before, having suppressed the menses, by an exposure to cold, had ever since, at irregular periods, suffered from hæmoptysis. When I saw her, which was in consultation, the hæmorrhage had already existed for forty-eight hours, the loss of blood very considerable, and she much exhausted. As the usual remedies had been unavailing, I induced Dr. Stewart, with whom I was attending, to try an emetic of ipecacuanha, which put an end to the hæmorrhage, and by proper management, subsequently, menstruation returned, and with it, a restoration of health.

Thirty years have since elapsed, during which lengthened period I have pursued this treatment, and with such success as to have inspired great confidence in it. Like all other means, it will sometimes fail, as might be expected, from the diversified nature of the causes and states of the hæmorrhage. But I am persuaded, when properly applied, it will be found to do more than any thing else,—and, certainly, from my own observations, it never produced mischief in the vital or spontaneous extravasations. The emetic I have preferred, and, indeed, only prescribed in these cases, is ipecacuanha.

To treat hæmoptysis in this mode, is not a practice resting on my authority exclusively.

Towards the middle of the last century, it was strenuously recommended by Dr. Bryan Robinson, of Dublin, whose publication on the subject attracted great attention. To his evidence in favour of it, might be added the attestations of several other respectable writers. Cullen, however, having tried the practice unhappily in a single case, probably from rupture, did much towards its condemnation, from the great weight of his authority. Neglected, it was not, however, entirely abandoned. We find, on the contrary, it receiving the support of Maryatt, Stoll, Burserius, Mosely, &c. Willis, too, so celebrated for his skill in the treatment of mania, especially for the cure of George III. of England, resorted to it freely, and declared that ample experience had taught him to confide in it above all other means, as well on account of its safety as efficacy. In this latter opinion, however, I do not entirely concur. Cases of hæmoptysis do occasionally arise from rupture, by ulceration of vessels, or mechanical causes, to which it is not at all adapted, and where, indeed, it might prove aggravatory, or even fatal, and such are not always readily discriminated.

The second indication in this weak form of hæmoptysis is to invigorate the system, and, through it, to impart tone to the relaxed or patulous vessels, and to rectify the state of the blood itself. To attain this end, the various astringents and tonics are usually called into requisition. Before prescribing any of them, it were well, however, to be assured by a careful perquisition, that no congestion or inflammation, or more serious lesions prevail. The state of hæmorrhage to which they are almost exclusively adapted, is where the process of hæmatosis is badly performed, or the system is rendered nearly exsanguineous by previous losses of blood from hæmorrhage, or in any other mode, that remaining being thin, pallid, and impoverished, oozing out chiefly from its own tenuity. Great disorder, or pravity of system, with the chlorotic or cachectic aspect, and extreme debility, are here observable.

The Peruvian bark was formerly among the first of the articles to attract attention. Many of the older writers confess its utility, and there are some who extravagantly praise it. But I seldom employ it, except in cases distinctly periodical in their nature, and here the sulphate of quinine is much to be preferred. It may be given alone, though its powers are sometimes improved by a combination with the chalybeates, of the efficacy of which much is asserted, and undoubtedly with justice. Eminently calculated are they to improve the constitution of the blood itself, and hence their utility in that species of hæmorrhage, owing to this condition chiefly. There is, however, a choice among the martial preparations. The muriated tincture is said to be best, though the hydriodate, the carbonate, the sulphate or phosphate of iron, answer very well, and especially the last.

Of the management of the idiopathic and more regular forms of hæmoptysis, I have now disposed. But it has anomalies originating in some peculiarity of cause, which ought not entirely to escape notice. Most of such cases are of a secondary nature, and were pointed out in tracing the etiology of hæmoptysis. Emanating from the irritation of a tubercular or any other essential pulmonary lesion, or derangement of the heart, or of the abdominal viscera, the effusion of blood, incidental only, must be subordinate to the pre-existing pathological condition, in every rational or efficient scheme of cure. But the consideration of these primary affections were alien to my present purpose, it being reserved for the future. Nor can I, with propriety, do more in this place, than summarily to state, that when hæmoptysis is owing to a suppression of the hæmorrhoidal, catamenial, or other discharge, or the repercussion of cutaneous eruptions, or the metastasis of gout or rheumatism, the endeavour should be to re-establish these several affections, in their original positions, and then to aim at their eradication,—or, excited by an elongation of the uvula, or enlarged tonsils, or any affection removable by a surgical operation, this is, at once, to be performed.

By prosecuting a course such as I have laid down, correctly shaping it to the peculiarities of each case, we shall frequently succeed in accomplishing a cure. Yet in some instances, and especially when it is connected with constitutional or local imperfection, hæmoptysis leaves behind it a liability to relapse on the slightest provocation. To guard against these repetitions of attack, a system of prophylactic instructions should be carefully suggested, and undeviatingly observed.

1st. The exciting causes of the hæmorrhage must be pointed out and avoided. Taking cold is the most common of these, and at the same time, is very apt to entail serious consequences. But there are others scarcely less to be apprehended, and among which are inordinate exertions of the voice. Let those especially, who are necessitated to pursue a profession dependent on public speaking, be impressed with the importance of moderating its tone. As one of many examples of the utility of this advice, we learn that Atticus, the friend of Cicero, having acquired the habit of vociferation, and suffering consequently from hæmoptysis, repaired to Athens, to be taught a more tempered and graceful elocution, in which succeeding, he had an exemption afterwards from the affection.

2d. In regard to regimen, some distinction is to be made, and first as to diet. To the active form of the hæmorrhage, vegetable matter, particularly the mucilaginous or farinaceous, is best suited; and to the other, light animal nutriment, as milk and eggs; and I have known malt liquors, in moderation, sometimes to prove very serviceable. Exercise, in each instance, is of importance, provided it be cautiously used, and the system properly prepared for it. On this point great errors are committed. Not unusually, patients are ordered on horseback, or even sent on a journey, with activity of pulse and febrile excitement. From such mal-practice, a recurrence of the hæmorrhage, with aggravation, must inevitably result.

3d. To watch the state of the pulse and respiration. Either thoracic pain or oppression, or any considerably increased force of circulation, is a sufficient ground of apprehension, and must be removed without delay. To effect the purpose, small bleedings, general or topical, are demanded, a still lower diet, a state of rest for the time, some laxative, or perhaps febrifuge medicine, and, in short, the whole antiphlogistic plan in all its parts. Where a slight hæmoptysis is attended by a quick and irritated pulse, and considerable mobility and weakness, digitalis has been found useful. No longer admissible is the loss of blood, and that article may be resorted to as a substitute, so administered, as just to affect the circulation, and keep it within its natural standard.

4th. Great good has been experienced from a succession of blisters, and these, where there is a considerable topical affection, are to be applied to the chest: under other circumstances, they will do very well on the extremities, acting as divellents. It is to be borne in mind, that even in less active hæmoptysis, though there be general debility, *local congestion*, with sometimes inflammation, may exist; and as the removal of these states is of primary importance, the appropriate remedies, though *depletory*, are not to be timidly withheld.

5th. In some very obstinate cases, a slight mercurial impression should be tried. The effect thus induced in the mouth serves, it has been said, as a diverticulum to the diseased action of the lungs. But more probably, by the general and revolutionary operation of mercury on the system, it supplants the disease, substituting its own peculiar action in place of it. To those cases in any degree connected with obstruction of the chyloporitic viscera, it is particularly adapted. An exception to this practice is to be found in a tubercular state of the lungs, or vitiation of the blood, formerly described, with which the use of mercury is utterly incompatible.

6th. Emetics, occasionally repeated, are entitled to confidence. They operate, by breaking up the habits and associations which continue the predisposition, and are, also, well calculated to emulge loaded vessels, and to distribute the blood equally throughout the circulation.

Conduct, however, the treatment as we may, hæmoptysis sometimes presents itself, of a nature so stubborn, that it will resist all these endeavours. Consulted, in such cases, we should advise, as the very last resource, a removal to a temperate climate, and by a voyage, when practicable. This has very often protracted life, and even effected permanent relief, where every thing else had failed, and under circumstances the least promising.

Of the treatment of hæmoptysis, I have only a few words more to say, and these regard the conduct of the case during, and immediately following, the flow of blood.

1st. The moment we are called to it, a state of rest, in bed, is to be enjoined, with the shoulders elevated, and the lower extremities extended, for reasons before stated.

2d. The chamber is to be kept cool, and well ventilated.

3d. Company should be excluded, and the patient not permitted to talk.

4th. Diet to consist of small portions of demulcent drinks, acidulated, and drank cold. It is right that the stomach be not loaded, as through it the lungs become oppressed.

5th. The bowels to be kept in a soluble state.

Not the least of the errors committed in the management of this disease, is an attention too exclusive to the mere suppression of the bleeding. The fact is, as previously stated, that such hæmorrhages are, for the most part, the efforts of nature to exonerate the lungs of oppressive accumulations of blood, or to reduce phlogosis, and, if not excessive, are probably as salutary as epistaxis in the affections of the brain. They may, it is true, leave some coagula or clots in the bronchial or cellular structure, which sometimes do harm, and this seems to me to be the main objection to permitting the effusion to continue. These remarks obviously apply to hæmorrhage of the mucous membrane only. It is very different with respect to the other form of the disease, where the consequences are so serious, that it should be arrested as speedily as possible. Cases, however, of this kind are so rare, that the principle is scarcely affected. No sound practitioner doubts, that the hæmorrhage of itself is comparatively of little moment, the real object of attention being the correction more especially of the morbid state of the pulmonary organs giving rise to it, and which, if not timely arrested, results too frequently in the full establishment of phthisis, or some other fatal lesion of the lungs.

Contemplated in another light, the ordinary treatment of this affection seems to me to be amenable to criticism. Governed by no principle, pathological or therapeutic, it is empirical, or at least tentative, every sort of nostrum or specific being tried to suppress the effusion of blood. Genuine hæmorrhage may be mostly resolved into one of two conditions, either inflammatory or congestive, and to be managed accordingly, whatever removing these states being the best calculated to put an end to the effusion, which is merely an effect. Guided by this view, and having little confidence in those articles usually deemed peculiarly appropriate, such as astringents, I seldom resort to them, preferring to conduct the cure on common principles, and by common remedies.

